# Effects of nonpharmacological interventions on the psychological health of high-risk pregnant women: a systematic review and meta-analysis

**DOI:** 10.4069/kjwhn.2021.09.17

**Published:** 2021-09-30

**Authors:** Hyeji Yoo, Sukhee Ahn

**Affiliations:** College of Nursing, Chungnam National University, Daejeon, Korea

**Keywords:** Anxiety, Gestational diabetes, High-risk pregnancy, Mental health, Premature obstetric labor

## Abstract

**Purpose:**

This study aimed to summarize the current evidence on the effects of nonpharmacological interventions on psychological health outcomes for women with high-risk pregnancies due to conditions such as preeclampsia, gestational diabetes, or preterm labor.

**Methods:**

The following databases were searched from January 2000 to December 2020: PubMed, Ovid Embase, CINAHL, Web of Science, DBpia, RISS, and KISS. Two investigators independently reviewed and selected articles according to the inclusion/exclusion criteria. RoB 2 and the ROBINS-I checklist were used to evaluate study quality.

**Results:**

Twenty-nine studies with a combined total of 1,806 pregnant women were included in the systematic review and meta-analysis. Psychological health improvements were found in women with preeclampsia (Hedges’ g=–0.67; 95% confidence interval [CI], –0.91 to –0.44), gestational diabetes (Hedges’ g=–0.38; 95% CI, –0.54 to –0.12), and preterm labor (Hedges’ g=–0.73; 95% CI, –1.00 to –0.46). The funnel plot was slightly asymmetrical, but the fail-safe N value and the trim-and-fill method showed no publication bias.

**Conclusion:**

Nonpharmacological interventions for women with high-risk pregnancies due to conditions such as preeclampsia, gestational diabetes, and preterm labor can improve psychological parameters such as anxiety, stress, and depression. Nurses can play a pivotal role in the nursing management of pregnant women with high-risk conditions and apply various types of nonpharmacological interventions to meet their needs in uncertain and anxious times during pregnancy.

## Introduction

High-risk pregnancy refers to a pregnancy that can threaten the health and life of the pregnant woman, fetus, or newborn [1]. Although precise statistical data for high-risk pregnancies have not been published, the number of women hospitalized for high-risk pregnancies increased by 3.5 times from 21,000 in 2006 to 77,000 in 2016 in Korea [2]. The proportion of premature births alone increased from 4.9% in 2006 to 7.7% in 2018 in Korea [1]. These findings indicate that high-risk pregnancies are continuing to become more common. Among the conditions that cause high-risk pregnancies, preeclampsia, gestational diabetes mellitus (GDM), and preterm labor (PTL) before 34 weeks of pregnancy are the most common [2]. The Korean Society of Obstetrics and Gynecology classifies these three as factors that cause a moderate- or higher-risk pregnancy and notes that intensive observation and management are necessary during pregnancy or childbirth for women with these conditions [1].

High-risk pregnant women often have negative experiences due to pharmacological treatment, restriction of physical activity, and hospitalization [3]. High-risk pregnant women are also more likely to be affected by poor psychological health such as depression, anxiety, and stress than other pregnant women [4-7]. In high-risk pregnant women in Korea, the prevalence of anxiety is 16%–34% and that of depression is 7%–33.9% [8]; similar rates have been reported among high-risk pregnant women in Western countries. Depression in high-risk pregnant women aggravates anxiety and stress [8] and negatively affects pregnancy maintenance and postpartum depression [6].

Some high-risk pregnant women are hospitalized in maternal-fetal intensive care units. This results in being separated from their spouse and family members [3], which may aggravate their anxiety, depression, and stress [9]. In addition, high-risk pregnant women have been reported as being less aware of the need for psychological health management than their low-risk counterparts [9].

Many intervention studies have been conducted for high-risk pregnant women. However, those studies mainly focused on changes in physical health indicators [10-12], including reductions in blood pressure [12], improvement of blood glucose levels [13], postpartum weight loss [11], and lowering of blood glucose levels in newborns [14]. Most systematic reviews of intervention studies on pregnant women with PTL only confirmed the treatment effect by applying drugs, tests, or treatment guidelines to prevent premature birth [10,15,16].

In recent years, increasingly many intervention studies have been conducted to improve aspects of psychological health such as depression, anxiety, and stress in high-risk pregnant women. These include relaxation therapy for pregnant women with preeclampsia [12] and face-to-face educational interventions combined with cognitive behavioral therapy and acupressure for pregnant women with GDM [17], which were found to effectively reduce stress. In addition, health care interventions for pregnant women with GDM were effective in relieving depression and anxiety [17]. Previous studies have reported the effects of interventions on psychological health by approaching high-risk pregnant women from the standpoint of disease. Still, no systematic review of nonpharmacological interventions effective for psychological health in high-risk pregnant women has not yet been reported. It is necessary to identify the evidence applicable in practice for the effects of interventions on psychological health, including anxiety, depression, and stress of high-risk pregnant women through a systematic review of the literature.

This study was conducted to confirm the effectiveness of nonpharmacological interventions applied to pregnant women experiencing preeclampsia, GDM, or PTL as high-risk conditions. The specific goals were as follows: first, to identify nonpharmacological interventions for pregnant women experiencing preeclampsia, GDM, or PTL; and second, to present a meta-analysis of the effects of nonpharmacological interventions on anxiety, depression, and stress.

## Methods

Ethics statement: This study was exempted from approval by the Institutional Review Board as it is a review of the literature using previously published studies.

### Study design

This study is a systematic review and meta-analysis. It was described according to the PRISMA (Preferred Reporting Items for Systematic Reviews and Meta-Analysis) 2020 guidelines [18].

### Criteria for selection of literature

The review questions were set using the PICO-SD (participants, intervention, comparison, outcome, study design) framework for a study that applied nonpharmacological interventions to pregnant women experiencing high-risk pregnancies and confirmed their effects. Studies were searched and selected from the electronic database.

#### Selection criteria

• Participants: The participants were pregnant women diagnosed with preeclampsia, GDM, or PTL as a high-risk condition.

• Intervention: All nonpharmacological interventions performed prenatally for the above-mentioned high-risk conditions during pregnancy were included.

• Comparison: Receiving routine antenatal care without nonpharmacological interventions during pregnancy.

• Outcomes: Anxiety, depression, or stress level.

• Study design: Only randomized controlled trials (RCTs) and non-RCTs using nonpharmacological interventions for high-risk pregnant women were included.

#### Exclusion criteria

The following types of studies were excluded: survey studies, qualitative studies, literature reviews, studies in which effect sizes could not be calculated, and studies presented at conferences.

### Literature search and collection

#### Literature search

The terms expressing interventions were identified through PubMed’s MeSH database before the literature search. The start time of the search was not limited, and the search site updated the literature by December 31, 2020. A total of seven search databases were used. PubMed, CINAHL (Cumulative Index to Nursing & Allied Health Literature), Web of Science, and Embase were used as international databases. In addition, RISS (the Korea Education and Research Information Service), KISS (Korea Research Information Service), and DBpia were used as domestic databases to identify studies published in Korea.

The search terms in the database were ‘preeclampsia,’ ‘GDM,’ ‘premature labor,’ ‘anxiety,’ ‘depression,’ and ‘stress,’ in combinations with ‘intervention.’ The search term for each risk was inputted as “TX (text) preeclampsia” OR “TI (title) preeclampsia” OR “AB (abstract) preeclampsia” OR “SU (subject) preeclampsia.” GDM and PTL were searched in the same way. All three search expressions were integrated. For the “intervention” search, the following terms were used “TX intervention” OR “TI intervention” OR “AB intervention” OR “SU intervention.” As terms related to the psychological health results, three search formulas were integrated by generating “TX anxiety” OR “TI anxiety” OR “AB anxiety” OR “SU anxiety” for anxiety, depression, and stress, respectively (Supplementary Data 1).

#### Data collection and selection

A list of documents collected through the literature search was generated. Using EndNote X9 (Clarivate Analytics, London, UK), a bibliographic management program, duplicate documents were removed from the list. The title and abstract of each study were checked to confirm whether the study met the data selection criteria. If it was difficult to decide whether to select a study based on the title and abstract, the full text of the study was reviewed. Two researchers (Yoo H and Ahn S) chose articles, discussed the results of selection, examined the content, and confirmed the final target literature.

#### Literature quality evaluation

The quality of the literature was evaluated independently by the two researchers using the revised Cochrane Risk of Bias tool for randomized trials (RoB 2) [19] and the Risk of Bias in Non-randomized Studies of Interventions (ROBINS-I) tool [20]. RoB 2 provides a framework for judging bias in the results of various types of randomized experimental studies [19]. RoB 2 consists of five subdomains: (1) bias due to the randomization process, (2) bias due to deviations from intended interventions, (3) bias due to missing outcome data, (4) bias in measurement of the outcome, and (5) bias in selection of the reported result. ROBINS-I is a tool to evaluate the non-randomized effects of interventions and compare two or more interventions [20]. ROBINS-I consists of seven subdomains: (1) bias due to confounding, (2) bias in selection of participants into the study, (3) bias in classification of interventions, (4) bias due to deviation from intended interventions, (5) bias due to missing data, (6) bias in measurement of outcomes, and (7) bias in selection of the reported result. Two researchers evaluated the literature quality individually, and reevaluated inconsistencies through consensus after reviewing the full text.

### Data analysis method

#### Characteristics of the literature

The characteristics of the selected studies were extracted using the framework of the following 12 items: age, pregnancy period, classification of high-risk pregnancy, sample size, intervention name, number of interventions, duration of interventions, dependent variables, research tools, research design, research results, and country.

#### Comprehensive effect size analysis

The effect size and homogeneity of nonpharmacological interventions were analyzed using the Comprehensive Meta-Analysis program. The mean and standard deviation or frequency were selected, and a random-effect model was applied to calculate the effect size for the results. The direction of the effect values of individual studies and the degree of overlap of the confidence intervals between studies were confirmed through forest plots. For statistical heterogeneity in the effect size, the chi-square test and I^2^ index were calculated. A higher value of the I^2^ index corresponds to greater heterogeneity: 0% means no heterogeneity; 50%, moderate heterogeneity; and 75% or more, high heterogeneity [21].

#### Publication bias test

The publication bias of the selected study was tested by the Egger linear regression asymmetry test [22], the fail-safe N coefficient [23], and the trim-and-fill method [24,25], including a funnel plot.

## Results

### Final literature selection

**Figure 1.**
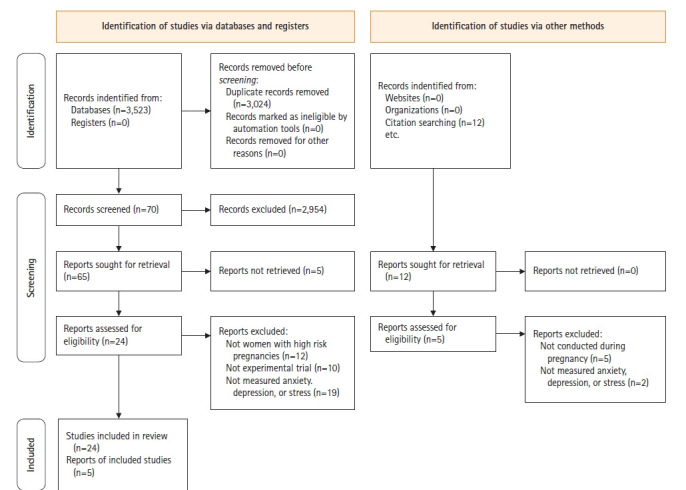
PRISMA 9Preferred Reporting Items for Systematic Reviews and Meta-Analysis) 2020 flow chart for the literature review.

Through the search, a total of 3,535 documents were first selected, and after excluding duplicate documents, 3,024 articles remained. Among them, 70 papers were selected by reviewing the titles and abstracts. The full text of these 70 papers was checked, and finally, 29 articles were selected for analysis [26-54]. Of the 41 articles excluded, 12 did not include preeclampsia, GDM, or PTL, 10 were non-experimental studies, and 19 did not have anxiety, depression, or stress as outcome variables (Figure 1).

### Quality evaluation of selected studies

**Figure 2.**
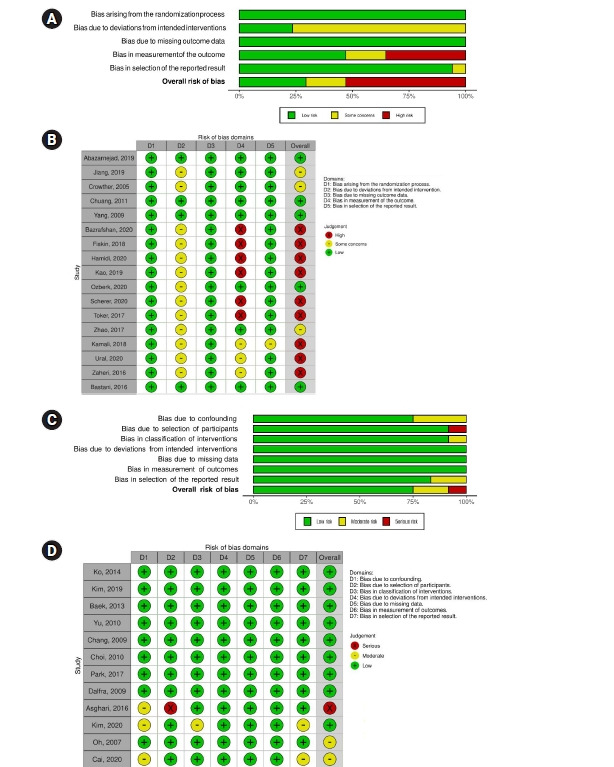
Risk of bias graphs for 17 randomized controlled trials (RCTs) (A, B) and 12 non-RCT studies (C, D). (A, C) Risk of bias summary. (B, D) Risk of bias for selected studies.

Quality evaluation using RoB 2 was performed for the 17 RCTs [26-42]. The risk of bias was low for both bias due to the randomization process and bias due to missing outcome data. Bias due to deviations from intended interventions showed some concern for about 76% of studies. The risk of bias in measurement of outcomes was as follows: 35%, high-risk of bias; 29%, low risk; 18%, moderate risk; and 53%, very high risk. An analysis of the methodological quality of the 12 non-RCT studies [43-54], using ROBINS-I found low risks of bias due to deviations from intended interventions, bias due to missing outcome data, and bias in measurement of outcomes. The risk of bias in the allocation process was moderate in 25% of papers. The risk of bias due to deviations from intended interventions was severe in 8% of studies. The risk of bias due to missing outcome data was moderate in 8% of studies. The risk of bias in selection of the reported result was moderate in 17% of the studies. Overall, 75% of the studies had a low risk of bias, 16% had a moderate risk, and 8% had a severe risk (Figure 2).

### General characteristics of selected studies

#### Research characteristics

• Country: Of the 29 studies, nine were conducted in Korea [43-46,48,50,52-54], all of which were non-RCT studies. Seven studies [27-29,33,35,41,47] were conducted in Iran. There were four studies each in China [26,34,42,49] and Turkey [32,37,39,40], and two each in Taiwan [30,36] and Switzerland [38]. One study was done in Australia [31] and one in Italy [51] (Supplementary Table 1).

• Year of publication: Of the 29 selected studies, seven [26,31,45,46,50,51,54] were published between 2005 and 2010. Three studies [30,48,53] were published between 2011 and 2014. The majority of studies (n=18) were published between 2016 and 2020 [27-29,32-37,39-44,47,49,52] (Supplementary Table 1).

#### Subject characteristics

• High-risk pregnancies: Of the 29 studies, five studies [28,34,35,39,47] included women with preeclampsia. There were 11 studies [27,31-33,40,41,48,49,51-53] targeting GDM and 11 studies [26,29,30,35-38,45,46,50,54] on PTL. Two studies [42,43] were conducted on women with high-risk pregnancies including PTL, GDM, and preeclampsia (Supplementary Table 1).

• Age: The age of the subjects was reported in 21 studies [27-34,36-38,42,43,45,46,48-52,54]. With the exclusion of one study [28], which presented only the age range, the average age of the subjects was 32.1 years and at least 17 years [28] (Table 1).

• Pregnancy period: The gestation period in the 29 articles ranged from 16 weeks [31] to 41 weeks [49] (Table 1).

#### Characteristics of interventions

• Research design: Of the 29 studies, 17 [26-42] were RCTs. Of the remaining 12 non-RCTs [43-54], seven studies used a nonequivalent control group pre- and posttest design [45,48,49,51-53], and four studies used a nonequivalent control group non-synchronized design [43,44,50,54]. One study had a matching control group interrupted time series design [46] (Supplementary Table 1).

• Sample size: All 29 studies had one experimental group and one control group, and the average sample size was 75 people each. The smallest sample size was 17 in the experimental and 18 in the control group [44], and the largest sample size was 490 in the experimental group and 510 in the control group [31] (Table 1).

• Intervention strengths: Except for four studies [31,34,49,51], which did not clearly report the details, the number of interventions could be confirmed. The intervention programs were provided an average of 7.7 times, with a range from at least one time [30] up to 30 times [31]. Except for four studies that did not report the relevant data [31,34,42,49], the average duration of the intervention was identified as 19.5 days. In both RCTs and non-RCTs, on average 7.7 interventions were provided for 19.6 days (Table 1).

• Types of intervention and consequences: The interventions were classified into education, counseling, and behavioral therapy. Studies that performed only one type were classified as monotherapy. (1) Education: Three studies conducted education, of which two studies [35,42] conducted psychological education. One study [45] provided high-risk pregnancy education. The educational intervention effectively relieved stress [35,45] and postpartum depression [42]. (2) Counseling: One study involved dietary counseling [31] and the other focused on blood glucose control [51]. Counseling was effective for depression at 3 months postpartum [31]. (3) Behavioral interventions: Twelve studies implemented behavioral therapy. Three studies [32,46,54] used abdominal breathing, three studies [26,39,44] used music therapy, and three studies [38,41,47] used cognitive behavioral therapy. Two studies [30,50] used relaxation therapy. Finally, one study [27] used acupressure therapy. Overall, behavioral therapy was effective in relieving anxiety [26,27,30,32,38,46,47,50,54], depression [32,47], and stress [30,32,38,41,44,47,50,54] (Supplementary Table 1).

Studies that offered two or more intervention approaches were classified as combination therapy. (1) Studies using education and counseling therapy: Of the seven studies, two studies [29,34] mixed high-risk pregnancy education and psychological counseling and four studies [48,49,52,53], mixed high-risk pregnancy education and lifestyle counseling. Only one study [40] conducted high-risk pregnancy management education and counseling. These combination interventions were effective in relieving anxiety [29,34,48,49,52] and depression [29,48,49,52,53]. (2) Studies using education and behavioral therapy: One study [28] used high-risk pregnancy education and anxiolytic therapy and another [33] used high-risk pregnancy education and breathing therapy. Education and behavioral therapy were effective in relieving anxiety [28,33]. (3) Studies using counseling and behavioral therapy: One study [36] used psychological counseling and conversion therapy, and another [37] used psychological counseling and relaxation therapy. Counseling and behavioral therapy were effective in relieving anxiety and depression [36]. (4) Combining education, counseling, and behavioral therapy: One study [43] found that this combination was effective in relieving depression (Supplementary Table 1).

• Intervention duration: Behavioral therapy can be divided into short-term interventions and long-term interventions. Considering that high-risk pregnant women are stable after 3 days of hospitalization [30], and women with PTL are hospitalized for 5 days on average [50], short-term interventions were defined as those conducted within a week. Short-term interventions included applying relaxation therapy for 1 to 5 days for pregnant women with PTL [30,50], providing music therapy for 3 to 4 days [26,44], applying abdominal breathing for three days [46,54], and performing acupressure for 3 days in pregnant women with GDM [27]. Long-term interventions (longer than a week) included cognitive behavioral therapy for 3 to 6 weeks for pregnant women with PTL [38], preeclampsia [47], and GDM [41] and abdominal breathing for a month for women with GDM [32]. In particular, abdominal breathing effectively relieved stress, anxiety, and depression [32]. Abdominal breathing also effectively reduced anxiety [46,54] and stress [54] in pregnant women in PTL. Abdominal breathing can be easily applied in clinical practice, and we therefore suggest that behavioral interventions applying abdominal breathing be actively used to improve the psychological health of high-risk pregnant women. Cognitive behavioral therapy showed improvement in stress [41] in pregnant women with GDM, alleviated depression, anxiety, and stress [47] in pregnant women with preeclampsia, and reduced anxiety and stress [38] in pregnant women with PTL. Therefore, expanding the use of cognitive behavioral interventions in clinical practice would have the benefits of reducing anxiety, depression, and stress in high-risk pregnant women.

• Intervention place: Nonpharmacological interventions were mainly provided as in-hospital interventions, including all five interventions for pregnant women with preeclampsia [28,34,35,39,47], and 10 out of 11 interventions for pregnant women with PTL [26,29,30,36,37,44-46,50,54]. Pregnant women with preeclampsia and PTL received pharmacological therapy during hospitalization and therapeutic management for ongoing monitoring of the pregnant woman and fetus after hospital discharge [3]. In pregnant women with GDM, seven out of 11 interventions [27,31-33,40,41,48,49,53] were applied in outpatient clinics and two interventions [27,49] were conducted in the hospital ward. This was most likely because providing integrated education and counseling on GDM self-management, such as blood glucose checks, lifestyle improvement, and drug treatment is possible in outpatient clinics.

• Measurement tools: Nine tools were used in 21 studies to measure anxiety: the Spielberger State-Trait Anxiety Questionnaire, the most popular tool, was used in 11 studies [26,28,30,31,33,38,39,43,50,52,54]; followed by the Depression Anxiety Stress Scale [32,35,41], visual analog scale [27,46,50], Hospital Anxiety and Depression [45,47], Beck Anxiety Inventory [36,48], Self-rating Anxiety Scale [34], Pregnancy-Related Anxiety [30], Pregnancy-Related Anxiety Questionnaire [29], and Maternal Anxiety Questionnaire [27]. For depression, seven instruments were used in 12 studies: the Self-Rating Depression Scale [34,52,53], the Edinburgh Postnatal Depression Scale [31,36,42], the Depression Anxiety Stress Scale [32,35,41], the Center for Epidemiologic Studies-Depression Scale [40,51], Beck Depression Inventory [48], Hospital Anxiety and Depression [47], and the Postpartum Depression Screening Scale [42], which was administered after delivery. For stress measurements, eight instruments were used in 11 studies: The PTL Stress Scale [44,50,54], Depression Anxiety Stress Scale [32,35,41], Perceived Stress Scale [30,38], visual analog scale [30], Diabetes-related Stress Scale [51], Diabetes Health Distress Scale [51], Pregnancy Distress Questionnaire [47], and Prenatal Posttraumatic Stress Questionnaire [35].

Outcome variables were measured using well-established self-report instruments for anxiety, depression, and stress. In order to supplement these subjective indicators, five studies also reported pregnant women’s blood pressure [26,39,46,50], pulse [50], heart rate [26], respiration [26], body temperature [30,46,50], and oxygen saturation [46]. In these five studies, improvements in physiological indicators were shown by a decrease in systolic blood pressure [26,39,46,50] and diastolic blood pressure [26,39,50], a decrease in pulse or heart rate [26,50], body temperature [30,46,50], and oxygen saturation [46].

### Effects of nonpharmacological interventions on psychological health

The effect size of the nonpharmacological interventions for high-risk pregnant women was calculated for the overall results including anxiety, depression, and stress; for the results of each of the three variables; according to the type of high-risk pregnancy; and according to the study design. The effect size was calculated using a random-effect model. Some studies included multiple measurements of the outcome variable. Thus, the meta-analysis of effect size included 26 sets of measurements of anxiety from 21 studies, 11 sets of measurements of depression from 12 studies, and 13 sets of measurements of stress from 11 studies.

#### (1) Effects by type of high-risk pregnancy

##### ① Effects on subjects with preeclampsia

A. Effects on anxiety: Nonpharmacological interventions showed an effect size of Hedge’s g=–0.42 (SE=.28) for anxiety in pregnant women with preeclampsia, but it was not statistically significant (*p*=.142). The five studies on anxiety in pregnant women with preeclampsia were highly heterogeneous (Q=28.61, df=4, *p*<.001, I^2^=86.02) [21].

B. Effects on depression: Nonpharmacological interventions had an effect size of Hedge’s g=–0.75 (SE=.14) for depression in pregnant women with preeclampsia (*p*<.001). The two studies on depression in pregnant women with preeclampsia showed low heterogeneity (Q=0.13, df=1, *p*=.719, I^2^<.001) [21].

**Figure 3.**
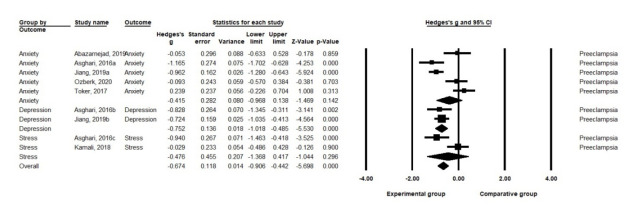
Effects of nonpharmacological interventions on pregnant women with preeclampsia.

C. Effects on stress: Nonpharmacological interventions showed an effect size of Hedge’s g=–0.48 (SE=.46) on stress in pregnant women with preeclampsia, which was not statistically significant (*p*=.296). The two studies on stress in pregnant women with preeclampsia showed moderate heterogeneity (Q=6.62, df=1, *p*=.010, I^2^=84.90) [21] (Figure 3, Supplementary Table 2).

##### ② Effects on subjects with GDM

A. Effects on anxiety: Nonpharmacological interventions had an effect size of Hedge’s g=–1.66 (SE=.46) on anxiety in pregnant women with GDM (*p*<.001). The eight studies reporting 10 sets of anxiety measurements in pregnant women with GDM were highly heterogeneous (Q=382.15, df=9, *p*<.001, I^2^=97.65) [21].

B. Effects on depression: Nonpharmacological interventions showed an effect size of Hedge’s g=–0.23 (SE=.11) for depression in pregnant women with GDM (*p*=.045). The seven studies reporting depression in pregnant women with GDM had a moderate level of heterogeneity (Q=11.85, df=6, *p*=.065, I^2^=49.37) [21].

**Figure 4.**
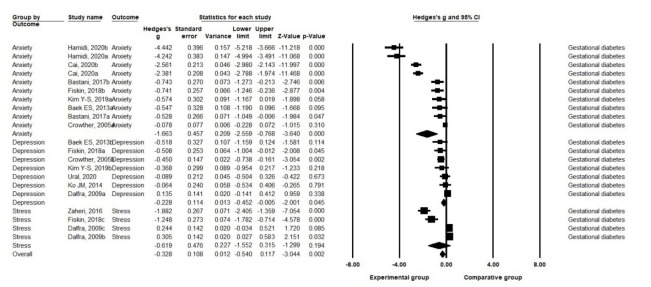
Effects of nonpharmacological interventions on pregnant women with gestational diabetes mellitus.

C. Effects on stress: Nonpharmacological interventions showed an effect size of Hedge’s g=–0.62 (SE=.48) for stress in pregnant women with GDM and were not statistically significant (*p*=.194). The three studies reporting four sets of stress measurements in pregnant women with GDM were highly heterogeneous (Q=76.30, df=3, *p*<.001, I^2^=96.07) [21] (Figure 4, Supplementary Table 2).

##### ③ Effects on subjects with PTL

A. Effects on anxiety: Nonpharmacological interventions showed an effect size of Hedge’s g=–0.97 (SE=.31) on the anxiety of pregnant women with PTL (*p*=.001). The eight studies reporting 10 sets of anxiety measurements were highly heterogeneous (Q=14.72, df=6, *p*=.023, I^2^=59.24) [21].

**Figure 5.**
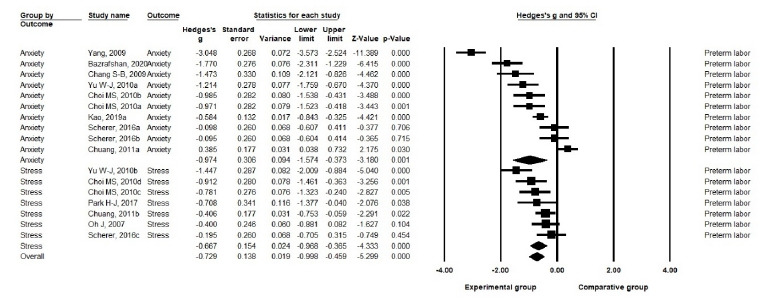
Effects of nonpharmacological interventions on pregnant women with preterm labor.

B. Effects on stress: Nonpharmacological interventions showed an effect size of Hedge’s g=–.75 (SE=.16) on the stress of pregnant women with PTL (*p*<.001). Six studies reporting seven sets of stress measurements in pregnant women with PTL showed a moderate level of heterogeneity (Q=11.66, df=5, *p*=.040, I^2^=57.11) [21] (Figure 5, Supplementary Table 2).

C. Only one of the PTL studies focused on depression.

#### (2) Effect size by study design

Details on effect size by RCT and non-RCT study design are presented in Supplementary Figures 2, 3.

##### ① Effect size for randomized trial design subjects

A. RCT effects on anxiety: Nonpharmacological interventions showed an effect size of Hedge’s g=–1.01 (SE=.26) on anxiety (I<.001). Of the 17 RCT studies, the 16 studies on anxiety were highly heterogeneous (Q=391.45, df=15, I<.001, I^2^=96.17) [21].

B. RCT effects on depression: Nonpharmacological interventions showed an effect size of Hedge’s g=–0.52 (SE=.08) on depression in randomized experimental design subjects (*p*<.001). The six studies on depression showed low heterogeneity (Q=6.73, df=5, *p*<.001, I^2^=25.69) [21].

C. RCT effects on stress: Nonpharmaceutical interventions showed an effect size of Hedge’s g=–0.77 (SE=.28) on subjects’ stress in randomized studies (*p*=.005). The six studies on stress showed high heterogeneity (Q=39.24, df=5, *p*<.001, I^2^=87.26) [21] (Supplementary Tabl 2, Supplementary Figure 2).

##### ② Effect size for non-RCT design subjects

A. Non-RCT effects on anxiety: Nonpharmacological interventions showed an effect size of Hedge’s g=–1.14 (SE=.32) on anxiety (*p*<.001). Of the 12 non-RCT studies, the 10 on anxiety were highly heterogeneous (Q=125.75, df=9, *p*<.001, I^2^=92.84) [21].

B. Non-RCT effects on depression: Nonpharmacological interventions showed an effect size of Hedge’s g=–0.29 (SE=.19) on depression, but it was not significant (*p*=.136). The five studies on depression had an intermediate level of heterogeneity (Q=12.62, df=4, *p*=.013, I^2^=68.31) [21].

C. Non-RCT effects on stress: Nonpharmacological interventions showed an effect size of Hedge’s g=–0.49 (SE=.25) on stress (*p*<.001). The seven studies on stress were highly heterogeneous (Q=54.75, df=6, *p*<.001, I^2^=89.04) [21] (Supplementary Table 2, Supplementary Figure 3).

### Publication bias test

Funnel plots, the classic fail-safe N, and the trim-and-fill method were used to test for publication bias related to the effects of nonpharmacological interventions on psychological health of high-risk pregnant women. The funnel plot was visually asymmetrical (Supplementary Figure 1), and the significance level of the degree of asymmetry through the Egger regression test was *p*<.001. The safety factor (classic fail-safe N) was calculated. This parameter, which refers to the number of studies required to change the results of nonpharmacological interventions on psychological health, was 6,235. This value was greater than the 260 calculated from the standard 5k+10 formula [23]. The effect of errors on the results was checked through the trim-and-fill method. No additional study was required, and both the observed effect size and the corrected effect size were –0.80. Therefore, combining the above results, it can be concluded that the overall effect size was not affected by publication bias (Supplementary Figure 1).

## Discussion

### Key results

In this study, 29 nonpharmacological intervention studies for pregnant women experiencing high-risk pregnancies were reviewed, including the method of application, the content of the intervention, and intervention effects on anxiety, depression, and stress. Furthermore, the effect size of the outcome index was evaluated. As a result of the meta-analysis, nonpharmacologic interventions showed significant effects on individual indicators of anxiety, depression, and stress.

### Interpretation

The health problems caused by high-risk pregnancy require long-term therapeutic management to maintain pregnancy and give birth at full term [1]. As a result, pregnant women experience anxiety, depression, and stress due to the burden of self-management and uncertainty about their health [9]. This study’s results showed that nonpharmacological interventions provided for high-risk pregnant women had the largest effect size for anxiety, compared to depression or stress as individual variables.

Previous studies confirmed the effectiveness of nonpharmacological interventions on physiological health only for pregnant women with preeclampsia [12] or pregnant women with GDM [17]. However, the present study confirmed the effects of nonpharmacological interventions on specific psychological outcomes for women with high-risk pregnancies who had preeclampsia, GDM, and/or PTL. Nonpharmacological interventions showed the greatest effect on depression in women with preeclampsia. In contrast, for pregnant women with GDM, the impact on anxiety was most prominent, and for pregnant women with PTL, anxiety, and stress were reduced. Therefore, the active use of nonpharmacological interventions in clinical practice can help improve psychological health indicators in high-risk pregnant women and positively affect maternal-infant health.

Based on the findings of this study, the effectiveness of online-based interventions remains unclear. An online counseling intervention applied to pregnant women with GDM did not significantly improve depression or stress scores [51]. However, online-based self-management and counseling appeared to reduce anxiety and depression in pregnant women with GDM [52] and an online-based cognitive behavioral stress management program reduced anxiety and stress in pregnant women with PTL [38]. As the physical activity of high-risk pregnant women is restricted [9] and the number of studies is insufficient, future research is needed to confirm the effect of non-face-to-face interventions. In addition, due to the recent social distancing due to coronavirus disease 2019, some regions are operating or have plans to run non-face-to-face prenatal programs [55,56]. As such, more active use of information and communications technology-based mediations would be beneficial.

The most common intervention providers were nurses or midwives. This is most likely due to the fact that high-risk pregnant women receive focused inpatient or outpatient care and nurses and midwives are highly accessible and have a heightened understanding of high-risk pregnancy. The majority of the interventions (86.2%) were performed individually. Group-based interventions constituted 13.8% and group sizes ranged from as few as four [29] to as many as 12 women [42]. Because the treatment schedule for each high-risk individual is different and bed rest is required when hospitalized, more interventions appear to have been delivered on an individualized basis. However, a group intervention for 10 pregnant women with preeclampsia per group effectively alleviated anxiety, depression, and stress [47]. As group interventions have been shown to affect psychological health in women with high-risk conditions [42], group interventions may be a reasonable option if individual access is difficult.

### Limitations

Since the results of this study were limited to anxiety, depression, and stress, the findings cannot be applied to other mental health or psychosocial health outcomes, such as uncertainty or self-efficacy. Since the Cochrane Library was not included in the literature search, it is possible that some studies may have been missed. Because most women with high-risk pregnancies who are hospitalized require bed rest, prior studies may have been faced limitations in applying physical activities or behavioral interventions. Thus, the location where intervention is applied, the intervention type, and limited activities of women with high-risk pregnancies may have affected the results of this study. The risk of bias in measurement of the outcome was high in RCTs, and randomization and blinding were not sufficiently described. These are limitations when evaluating the quality of the studies. In non-RCT studies, the risk of bias in selection of participants into the study was deemed high because the criteria and process for selection were not clearly described. The fact that the outcome variables of anxiety, depression, and stress were measured using self-report questionnaires also increases the risk of bias in measurement of the outcome. If subject blinding is not performed, there is a possibility that the intervention effect can be overestimated. Therefore, caution should be considered when interpreting the non-RCT study results.

This study presented evidence regarding whether nonpharmacological interventions improve anxiety, depression, and stress in high-risk pregnant women with preeclampsia, GDM, and/or PTL. The effectiveness of face-to-face interventions was confirmed, but the impact of online-based interventions on psychological health remains unclear. When education, counseling, and behavioral therapy were applied as single or multiple interventions for high-risk pregnant women, their psychological health improved. Nurses need to apply these nonpharmacological interventions for women with high-risk pregnancies considering their nursing needs and the environment where the intervention is provided. In further research, the effect of online-based interventions will be checked using both self-reporting questionnaires and vital signs as much as possible.

## Figures and Tables

**Table 1.** Descriptive summary of the selected studies (N=29)

First author (year) [reference]	Country	Study design	Participant		Sample size		Intervention detail				Psychological outcome		
			Age (year)	Gestational weeks	Int	Cont	Content	Number of sessions	Time	Duration	Anxiety	Depression	Stress
Preeclampsia													
Abazarnejad (2019) [28]	Iran	RCT	17–46	25–38	22	22	Psychoeducational counseling	2	45	3 days	Int<Cont at T1	NA	NA
Jiang (2019) [34]	China	RCT	21–48 (31.7)	≥ 20	84	84	Comprehensive nursing intervention	ND	ND	ND	Int<Cont at T1	Int<Cont at T1	NA
Kamali (2018) [35]	Iran	RCT	ND	34–38	36	36	Face-to-face education in the health care system	3	45–60	3 days	NA	NA	Int<Cont at T1
Toker (2017) [39]	Turkey	RCT	ND	≥30	35	35	Classical Turkish music	7	30	7 days	None	NA	NA
Asghari (2016) [47]	Iran	Non-RCT	ND	28–34	31	30	Cognitive behavioral therapy	12	90	4 weeks	Int<Cont at T1	Int<Cont at T1	Int<Cont at T1
Gestational diabetes													
Hamidi (2020) [33]	Iran	RCT	29.6	24–28	44	44	Mindfulness-based training	8	120	8 weeks	Int<Cont at T1 & T2	NA	NA
Ural (2020) [40]	Turkey	RCT	ND	34–38	46	42	Health-promoting lifestyle education program	3	45	3 days	NA	None	NA
Fiskin (2018) [32]	Turkey	RCT	31.0	24–28 (27.2)	32	31	Diaphragmatic breathing exercise	30	15	30 days	Int<Cont at T1 & T2	Int<Cont at T1 & T2	Int<Cont at T1 & T2
Bastani (2016) [27]	Iran	RCT	29.9	ND	28	29	Acupressure	9	18	3 days	Int<Cont at T1	NA	NA
Zaheri (2016) [41]	Iran	RCT	ND	24–32	40	40	Cognitive behavioral stress management intervention training	6	120	3 weeks	NA	NA	Int<Cont at T1
Crowther (2005) [31]	Australia	RCT	30.5	16–30	490	510	Ongoing care including individualized dietary advice	ND	ND	ND	None at T1 & T4	Int<Cont at T4	NA
Cai (2020) [49]	China	Non-RCT	30.99	34–41	89	70	Comprehensive nursing mode	ND	ND	ND	Int<Cont at T1	Int<Cont at T1	NA
Kim (2019) [52]	Korea	Non-RCT	35.8	24–28	22	22	12-week web-based self-management program	12	20–30	12 weeks	Int<Cont at T1	Int<Cont at T1	NA
Ko (2014) [53]	Korea	Non-RCT	ND	24	34	34	Coaching program on comprehensive lifestyle modification	4	20–30	4 weeks	NA	Int<Cont at T1	NA
Baek (2013) [48]	Korea	Non-RCT	33	24–28	19	18	Case management program	5	ND	2 weeks	Int<Cont at T1	Int<Cont at T1	NA
Dalfrà (2009) [51]	Italy	Non-RCT	34.0	12–28	105	150	Telemedicine	ND	ND	10 weeks	NA	None	None
Preterm labor													
Bazrafshan (2020) [29]	Iran	RCT	26.1	24–28	36	36	Educational counseling group intervention	6	60	6 weeks	Int<Cont at T1 & T2	NA	NA
Özberk (2020) [37]	Turkey	RCT	27.9	29.8	33	33	Relaxation-focused nursing care	4	40–105	2 days	Int<Cont at T1, T2, & T3	NA	NA
Scherer (2016) [38]	Swiss	RCT	32.7	18–32 (28.4)	31	27	Internet-based cognitive behavioral stress management	6	ND	6 weeks	T1<T0 in Int	NA	T1<T0 in Int
Kao (2019) [36]	Taiwan	RCT	33.0	20–36 (27)	140	103	Support intervention	3–5	30–40	2 weeks	Int<Cont at T1	Int<Contat T1	NA
Chuang (2011) [30]	Taiwan	RCT	31.1	20–34	68	61	Relaxation training program	1	13	1 day	Int<Cont at T2	NA	Int<Cont at T1
Yang (2009) [26]	China	RCT	DK	28–36	60	60	Music therapy	3	30	3 days	T1<T0 in Int	NA	NA
Park (2017) [44]	Korea	Non-RCT	DK	20–37	17	18	Music therapy	12	15	4 days	NA	NA	Int<Cont at T1
Choi (2010) [50]	Korea	Non-RCT	32.0	20–38	29	26	Relaxation therapy	10	ND	5 days	Int<Cont at T1 & T2	NA	Int<Cont at T1 & T2
Yu (2010) [54]	Korea	Non-RCT	29.6	20–37	30	30	Abdominal breathing	9	ND	3 days	Int<Cont at T1	NA	Int<Cont at T1
Chang (2009) [46]	Korea	Non-RCT	31.5	24–37	26	20	Abdominal breathing	3	5	3 days	Int<Cont at T1, T2, & T3	NA	NA
Oh (2007) [5]	Korea	Non-RCT	20–36 (30.8)	20–36 (30.5)	33	33	Providing information	2	ND	10 days	NA	NA	T1<T0 in Int
High-risk pregnancy													
Kim (2020) [43]	Korea	Non-RCT	33.46	20–33	29	30	Supportive program	10	10–60	10 days	Int<Cont at T1	NA	NA
Zhao (2017) [42]	China	RCT	30.5	≥28	167	167	Couple-separated psychoeducational program for first-time parents	6	90	ND	NA	Int<Cont at T3	NA

Cont: Control group; Int: intervention group; NA: not applicable; ND: not described; RCT: randomized clinical trial; T1: time point 1; T2: time point 2; T3: time point 3.

## Supplementary materials

Further details on supplementary materials are presented online (available at https://doi.org/10.4069/kjwhn.2021.09.17).

Supplementary Data 1. Search terms used in the electronic databases

Supplementary Table 1. General characteristics and research methodology of the included studies (N=29)

Supplementary Table 2. Overall effect size of nonpharmacological interventions in women with high-risk pregnancies (N=29)

Supplementary Figure 1. Publication bias of selected studies.

Supplementary Figure 2. Effects of nonpharmacological interventions in randomized controlled trials (RCTs) on women with high-risk pregnancies.

Supplementary Figure 3. Effects of nonpharmacological interventions in non-randomized controlled trials (non-RCT) on women with high-risk pregnancies.
