# Supplementary material for: Effects of nonpharmacological interventions on the psychological health of high-risk pregnant women: a systematic review and meta-analysis
Source: Korean J Women Health Nurs. 2021 Sep 30;27(3):180–95. doi: 10.4069/kjwhn.2021.09.17 (PMC9328588; doi:10.4069/kjwhn.2021.09.17)
Supplement: Supplementary Data 1. — Search terms used in the electronic databases [file kjwhn-2021-09-17-suppl1.pdf]

Supplementary Data 1. Search terms used in the electronic databases

1. CINAHL

([“TX preeclampsia” OR “TI preeclampsia” OR “AB preeclampsia” OR “SU preeclampsia”] OR [“TX GDM” OR “TI GDM” OR “AB GDM” OR “SU GDM”] OR [“TX premature labor” OR “TI premature labor” OR “AB premature labor” OR “SU premature labor”] AND [“TX intervention” OR “TI intervention” OR “AB intervention” OR “SU intervention”] AND [“TX anxiety” OR “TI anxiety” OR “AB anxiety” OR “SU anxiety”] OR [“TX depression” OR “TI depression” OR “AB depression” OR “SU depression”] OR [“TX stress” OR “TI stress” OR “AB stress” OR “SU stress”])

2. PubMed

((“pre eclampsia”[MeSH Terms] OR “pre eclampsia”[All Fields] OR “preeclampsia”[All Fields] OR “preeclampsia”[Title] OR “pre-eclampsia”[Title/Abstract] OR “pre eclampsia”[MeSH Terms] OR (“diabetes, gestational”[MeSH Terms] OR (“diabetes”[All Fields] AND “gestational”[All Fields]) OR “GDM”[All Fields] OR (“gestational”[All Fields] AND “diabetes”[All Fields]) OR “GDM”[Title] OR “GDM”[Title/Abstract] OR “diabetes, gestational”[MeSH Terms]) OR (“obstetric labor, premature”[MeSH Terms] OR (“obstetric”[All Fields] AND “labor”[All Fields] AND “premature”[All Fields]) OR “premature obstetric labor”[All Fields] OR (“preterm”[All Fields] AND “labor”[All Fields]) OR “PTL”[All Fields] OR “PTL”[Title] OR “PTL”[Title/Abstract] OR “obstetric labor, premature”[MeSH Terms])) AND (“intervention s”[All Fields] OR “interventions”[All Fields] OR “interventive”[All Fields] OR “methods”[MeSH Terms] OR “methods”[All Fields] OR “intervention”[All Fields] OR “interventional”[All Fields] OR “intervention”[Title] OR “intervention”[Title/Abstract] OR “methods”[MeSH Terms]) AND (“depressed”[All Fields] OR “depression”[MeSH Terms] OR “depression”[All Fields] OR “depressions”[All Fields] OR “depression s”[All Fields] OR “depressive disorder”[MeSH Terms] OR (“depressive”[All Fields] AND “disorder”[All Fields]) OR “depressive disorder”[All Fields] OR “depressivity”[All Fields] OR “depressive”[All Fields] OR “depressively”[All Fields] OR “depressiveness”[All Fields] OR “depressives”[All Fields] OR “depression”[Title] OR “depression”[Title/Abstract] OR (“depressive disorder”[MeSH Terms] OR “depression”[MeSH Terms]) OR (“depressed”[All Fields] OR “depression”[MeSH Terms] OR “depression”[All Fields] OR “depressions”[All Fields] OR “depression s”[All Fields] OR “depressive disorder”[MeSH Terms] OR (“depressive”[All Fields] AND “disorder”[All Fields]) OR “depressive disorder”[All Fields] OR “depressivity”[All Fields] OR “depressive”[All Fields] OR “depressively”[All Fields] OR “depressiveness”[All Fields] OR “depressives”[All Fields] OR “depression”[Title] OR “depression”[Title/Abstract]) OR (“anxiety”[MeSH Terms] OR “anxiety”[All Fields] OR “anxieties”[All Fields] OR “anxiety s”[All Fields] OR “anxiety”[Title] OR “anxiety”[Title/Abstract] OR “anxiety”[MeSH Terms]))) AND ((ffrft[Filter]) AND (fft[Filter]))

3. Embase

#1 = (preeclampsia OR preeclampsia:ti OR preeclampsia:ab,ti OR preeclampsia:ab) AND [1966-2020]/py
#2 = (gestational AND diabetes OR ‘GDM’:ti OR ‘GDM’:ab,ti OR ‘GDM’:ab) AND [1966-2020]/py
#3 = (preterm AND labor OR ‘PTL’:ti OR ‘PTL’:ab,ti OR ‘PTL’:ab) AND [1966-2020]/py
#4 = (intervention OR intervention:ti OR intervention:ab,ti OR intervention:ab) AND [1966-2020]/py
#5 = (depression OR depression:ti OR depression:ab,ti OR depression:ab) AND [1966-2020]/py
#6 = (stress OR stress:ti OR stress:ab,ti OR stress:ab) AND [1966-2020]/py
#7 = (anxiety OR anxiety:ti OR anxiety:ab,ti OR anxiety:ab) AND [1966-2020]/py
#8 = #1 OR #2 OR #3
#9 = #5 OR #6 OR #7
#10 = #4 AND #8 AND #9
#11 = (preeclampsia OR preeclampsia:ti OR preeclampsia:ab,ti OR preeclampsia:ab) AND [1966-2020]/py2021-03-122021-03-1266235
#12 = (gestational AND diabetes OR ‘GDM’:ti OR ‘GDM’:ab,ti OR ‘GDM’:ab) AND [1966-2020]/py2021-03-122021-03-1236303
#13 = (preterm AND labor OR ‘PTL’:ti OR ‘PTL’:ab,ti OR ‘PTL’:ab) AND [1966-2020]/py2021-03-122021-03-1237000
#14 = (intervention OR intervention:ti OR intervention:ab,ti OR intervention:ab) AND [1966-2020]/py2021-03-122021-03-121022756
#15 = (depression OR depression:ti OR depression:ab,ti OR depression:ab) AND [1966-2020]/py2021-03-122021-03-12711320

#16 = (stress OR stress:ti OR stress:ab,ti OR stress:ab) AND [1966-2020]/py2021-03-122021-03-121342415
#17 = (anxiety OR anxiety:ti OR anxiety:ab,ti OR anxiety:ab) AND [1966-2020]/py2021-03-122021-03-12395243
#18 = #11 OR #12 OR #132021-03-122021-03-12125597
#19 = #15 OR #16 OR #172021-03-122021-03-122100169
#20 = #14 AND #18 AND #192021-03-122021-03-12565
#21 = #20 AND 'Article'/it2021-03-122021-03-12295
#22 = #20 AND 'article'/it AND ([english]/lim OR [korean]/lim)

4. Web of Science

1 = ((TS = (preeclampsia)) OR TI = (preeclampsia)) OR AB = (preeclampsia) OR KP = (preeclampsia)
2 = ((TS = (GDM)) OR TI = (GDM)) OR AB = (GDM) OR KP = (GDM)
3 = ((TS = (premature labor)) OR TI = (premature labor)) OR AB = (premature labor) OR KP = (premature labor)
4 = ((TS = (intervention)) OR TI = (intervention)) OR AB = (intervention) OR KP = (intervention)
5 = ((TS = (anxiety)) OR TI = (anxiety)) OR AB = (anxiety) OR KP = (anxiety)
6 = ((TS = (depression)) OR TI = (depression)) OR AB = (depression) OR KP = (depression)
7 = ((TS = (stress)) OR TI = (stress)) OR AB = (stress) OR KP = (stress)
8 = #1 OR #2 OR #3
9 = #5 OR #6 OR #7
10 = #4 AND #8 AND #9

5. DBpia, RISS, KISS

1) In Korean

[자간전증 AND 중재 AND 불안]
[자간전증 AND 중재 AND 우울]
[자간전증 AND 중재 AND 스트레스]
[임신성 당뇨 AND 중재 AND 불안]
[임신성 당뇨 AND 중재 AND 우울]
[임신성 당뇨 AND 중재 AND 스트레스]
[조기진통 AND 중재 AND 불안]
[조기진통 AND 중재 AND 우울]
[조기진통 AND 중재 AND 스트레스]

2) In English

[preeclampsia AND intervention AND anxiety]
[preeclampsia AND intervention AND depression]
[preeclampsia AND intervention AND stress]
[GDM AND intervention AND anxiety]
[GDM AND intervention AND depression]
[GDM AND intervention AND stress]
[premature labor AND intervention AND anxiety]
[premature labor AND intervention AND depression]
[premature labor AND intervention AND stress]

https://doi.org/10.4069/kjwhn.2021.09.17
