# Supplementary material for: Effects of nonpharmacological interventions on the psychological health of high-risk pregnant women: a systematic review and meta-analysis
Source: Korean J Women Health Nurs. 2021 Sep 30;27(3):180–95. doi: 10.4069/kjwhn.2021.09.17 (PMC9328588; doi:10.4069/kjwhn.2021.09.17)
Supplement: Supplementary Table 1. — General characteristics and research methodology of the included studies (N=29) [file kjwhn-2021-09-17-suppl2.pdf]

Supplementary Table 1. General characteristics and research methodology of the included studies (N=29)

Variable	Categories	(%)	Reference
Country	Korea	9 (31.0)	[43-46,48,50,52-54]
	Iran	7 (24.1)	[27-29,33,35,41,47]
	China	4 (13.8)	[26,34,42,49]
	Turkey	4 (13.8)	[32,37,39,40]
	Taiwan	2 (6.9)	[30,36]
	Swiss	1 (3.4)	[38]
	Italy	1 (3.4)	[51]
	Australia	1 (3.4)	[31]
Publication year	2005-2010	7 (24.1)	[26,31,45,46,50,51,54]
	2011-2014	3 (10.3)	[30,48,53]
	2016-2020	18 (62.1)	[27-29,32-37,39-44,47,49,52]
Study design	Randomized controlled trial	17 (58.6)	[26-42]
	Non-randomized controlled trial	12 (41.4)	[43-54]
	Nonequivalent control group pre- and posttest design	7 (24.1)	[45,47,48,49,51-53]
	Nonequivalent control group non-synchronized design	4 (13.8)	[43,44,50,54]
	Matching control group interrupted time series design	1 (3.5)	[46]
Intervention			
	Women with preeclampsia	5 (17.2)	[28,34,35,39,47]
	Women with gestational diabetes	11 (37.9)	[27,31-33,40,41,48,49,51-53]
	Women with preterm labor	11 (37.9)	[26,29,30,35-38,45,46,50,54]
	Women with high-risk pregnancies†	2 (6.9)	[42,43]
	Hospital or clinics	26 (89.7)	[26-37,39-50,53,54]
	Home	3 (10.3)	[38,51,52]
	Nurse or midwife	26 (89.7)	[26-37,39-46,48-50,52-54]
	Psychotherapist or psychologist	2 (6.9)	[38,47]
	Physician	1 (3.4)	[51]
	Individual	25 (86.2)	[26-28,30-41,43-46,48-52,54]
	Group	4 (13.8)	[29, 42, 47, 53]
	Face-to-face	26 (89.7)	[26-37, 39-50, 53, 54]
	Online-based	3 (10.3)	[38, 51, 52]
	Single	17 (58.6)	[26, 27, 30-32, 35, 38, 39, 41, 42, 44-47, 50, 51, 54]
	Multiple	12 (41.4)	[9, 28, 29, 33, 34, 36, 37, 40, 48, 49, 52-54]
	Education	13 (31.0)	[28, 29, 33-35, 40, 42, 43, 45, 48, 49, 52, 53]
	Counseling	12 (28.6)	[29, 31, 34, 36, 37, 40, 43, 48, 49, 51-53]
	Behavioral therapy	17 (40.5)	[26-28, 30, 32, 33, 36-39, 41, 43, 44, 46, 47, 50, 54]
	Education and counseling	7 (58.3)	[29, 34, 40, 48, 49, 52, 53]
	Education and behavioral therapy	2 (16.7)	[28, 33]
	Counseling and behavioral therapy	2 (16.7)	[36, 37]
	Education, counseling, behavioral therapy	1 (8.3)	[43]
Outcome variables‡	Anxiety	21 (47.7)	[26-34,36-39,43,46-50,52,54]
	Depression	12 (27.2)	[31,32,34,36,40,42,47-49,51-53]
	Stress	11 (25.0)	[30,32,35,38,41,44,45,47,50,51,54]

†Included preeclampsia, gestational diabetes, and preterm labor.
‡Duplication.
§Part of a combined intervention.
