# Supplementary material for: Effects of nonpharmacological interventions on the psychological health of high-risk pregnant women: a systematic review and meta-analysis
Source: Korean J Women Health Nurs. 2021 Sep 30;27(3):180–95. doi: 10.4069/kjwhn.2021.09.17 (PMC9328588; doi:10.4069/kjwhn.2021.09.17)
Supplement: Supplementary Table 2. — Overall effect size of nonpharmacological interventions in women with high-risk pregnancies (N=29) [file kjwhn-2021-09-17-suppl3.pdf]

Supplementary Table 2. Overall effect size of nonpharmacological interventions in women with high-risk pregnancies (N=29)

Variable	Categories	Outcome	n	Hedge's g	SE	Lower	Upper	*p*	Q	df	*p*	I^2
Type of high-risk pregnancy	Preeclampsia	Anxiety	5	−0.42	0.28	−0.97	0.14	0.142	28.61	4	< .001	86.02
		Depression	2	−0.75	0.14	−1.02	−0.49	< .001	0.13	1	0.719	< .001
		Stress	2	−0.48	0.46	−1.37	0.42	0.296	6.62	1	0.01	84.9
	Gestational diabetes	Anxiety	10	−1.66	0.46	−2.56	−0.77	< .001	382.15	9	< .001	97.65
		Depression	7	−0.23	0.11	−0.45	−0.01	0.045	11.85	6	0.065	49.37
		Stress	4	−0.62	0.48	−1.55	0.32	0.194	76.3	3	< .001	96.07
	Preterm labor	Anxiety	10	−0.97	0.31	−1.57	−0.37	0.001	151.65	9	< .001	94.07
		Stress	7	−0.67	0.15	−0.97	−0.67	< .001	14.72	6	0.023	59.24
Study design	RCT	Anxiety	16	−1.01	0.26	−1.52	−0.51	< .001	391.45	15	0.242	96.17
		Depression	6	−0.52	0.08	−0.67	−0.37	< .001	6.73	5	< .001	25.69
		Stress	6	−0.77	0.28	−1.31	−0.23	0.005	39.24	5	< .001	87.26
	Non-RCT	Anxiety	10	−1.14	0.32	−1.77	−0.52	< .001	125.75	9	< .001	92.84
		Depression	5	−0.29	0.19	−0.66	0.09	0.136	12.62	4	0.013	68.31
		Stress	7	−0.49	0.25	−0.99	< .001	0.051	54.75	6	< .001	89.04

RCT: Randomized controlled trial.
