# Supplementary figures and images for: Effects of nonpharmacological interventions on the psychological health of high-risk pregnant women: a systematic review and meta-analysis
Source: Korean J Women Health Nurs. 2021 Sep 30;27(3):180–95. doi: 10.4069/kjwhn.2021.09.17 (PMC9328588; doi:10.4069/kjwhn.2021.09.17)

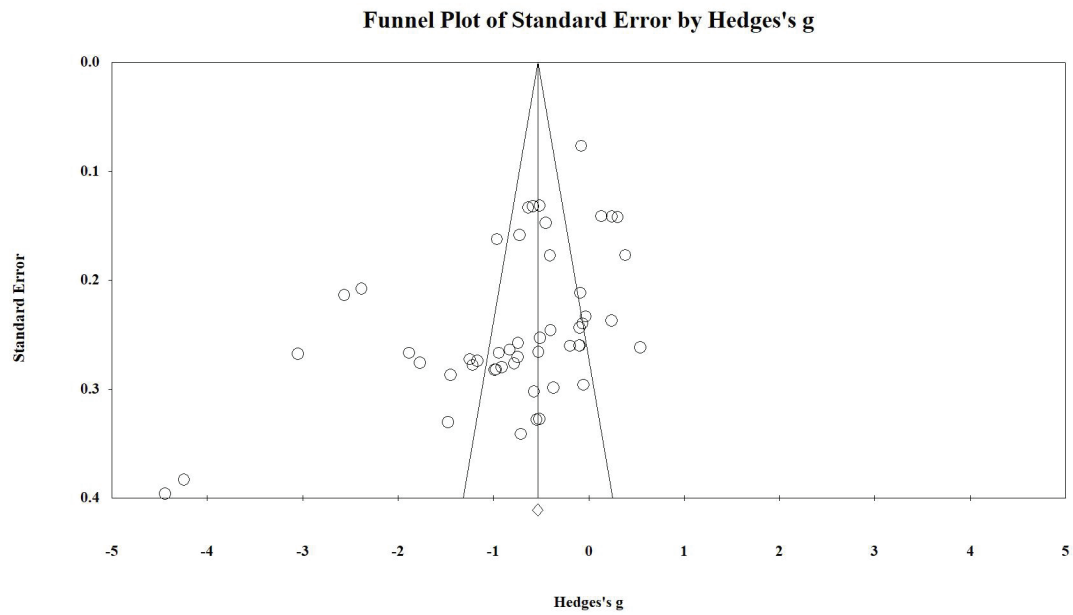

**Supplementary Figure 1.** Publication bias of selected studies.

Supplement: Supplementary Figure 1. — Publication bias of selected studies. [file kjwhn-2021-09-17-suppl4.pdf]
