# Supplementary material for: Effects of nonpharmacological interventions on the psychological health of high-risk pregnant women: a systematic review and meta-analysis
Source: Korean J Women Health Nurs. 2021 Sep 30;27(3):180–95. doi: 10.4069/kjwhn.2021.09.17 (PMC9328588; doi:10.4069/kjwhn.2021.09.17)
Supplement: Supplementary Figure 2. — Effects of nonpharmacological interventions in randomized controlled trials (RCTs) on women with high-risk pregnancies. [file kjwhn-2021-09-17-suppl5.pdf]

Group by Outcome	Study name	Outcome	Hedges's g	Standard error	Variance	Lower limit	Upper limit	Z-Value	p-Value	
Anxiety	Hamidi, 2020b	Anxiety	-4.442	0.396	0.157	-5.218	-3.666	-11.218	0.000	RCT
Anxiety	Hamidi, 2020a	Anxiety	-4.242	0.383	0.147	-4.994	-3.491	-11.068	0.000	RCT
Anxiety	Yang, 2009	Anxiety	-3.048	0.268	0.072	-3.573	-2.524	-11.389	0.000	RCT
Anxiety	Bazrafshan, 2020	Anxiety	-1.770	0.276	0.076	-2.311	-1.229	-6.415	0.000	RCT
Anxiety	Jiang, 2019a	Anxiety	-0.962	0.162	0.026	-1.280	-0.643	-5.924	0.000	RCT
Anxiety	Bastani, 2017b	Anxiety	-0.743	0.270	0.073	-1.273	-0.213	-2.746	0.006	RCT
Anxiety	Fiskin, 2018b	Anxiety	-0.741	0.257	0.066	-1.246	-0.236	-2.877	0.004	RCT
Anxiety	Kao, 2019a	Anxiety	-0.584	0.132	0.017	-0.843	-0.325	-4.421	0.000	RCT
Anxiety	Bastani, 2017a	Anxiety	-0.528	0.266	0.071	-1.049	-0.006	-1.984	0.047	RCT
Anxiety	Scherer, 2016a	Anxiety	-0.098	0.260	0.068	-0.607	0.411	-0.377	0.706	RCT
Anxiety	Scherer, 2016b	Anxiety	-0.095	0.260	0.068	-0.604	0.414	-0.365	0.715	RCT
Anxiety	Ozberk, 2020	Anxiety	-0.093	0.243	0.059	-0.570	0.384	-0.381	0.703	RCT
Anxiety	Crowther, 2005a	Anxiety	-0.078	0.077	0.006	-0.228	0.072	-1.015	0.310	RCT
Anxiety	Abazarnejad, 2019	Anxiety	-0.053	0.296	0.088	-0.633	0.528	-0.178	0.859	RCT
Anxiety	Toker, 2017	Anxiety	0.239	0.237	0.056	-0.226	0.704	1.008	0.313	RCT
Anxiety	Chuang, 2011a	Anxiety	0.385	0.177	0.031	0.038	0.732	2.175	0.030	RCT
Anxiety			-1.014	0.258	0.067	-1.520	-0.507	-3.922	0.000	
Depression	Jiang, 2019b	Depression	-0.724	0.159	0.025	-1.035	-0.413	-4.564	0.000	RCT
Depression	Zhao, 2017	Depression	-0.632	0.133	0.018	-0.893	-0.371	-4.740	0.000	RCT
Depression	Kao, 2019b	Depression	-0.513	0.131	0.017	-0.771	-0.255	-3.903	0.000	RCT
Depression	Fiskin, 2018a	Depression	-0.508	0.253	0.064	-1.004	-0.012	-2.008	0.045	RCT
Depression	Crowther, 2005b	Depression	-0.450	0.147	0.022	-0.738	-0.161	-3.054	0.002	RCT
Depression	Ural, 2020	Depression	-0.089	0.212	0.045	-0.504	0.326	-0.422	0.673	RCT
Depression			-0.517	0.077	0.006	-0.667	-0.366	-6.726	0.000	
Stress	Zaheri, 2016	Stress	-1.882	0.267	0.071	-2.405	-1.359	-7.054	0.000	RCT
Stress	Fiskin, 2018c	Stress	-1.248	0.273	0.074	-1.782	-0.714	-4.578	0.000	RCT
Stress	Asghari, 2016c	Stress	-0.940	0.267	0.071	-1.463	-0.418	-3.525	0.000	RCT
Stress	Chuang, 2011b	Stress	-0.406	0.177	0.031	-0.753	-0.059	-2.291	0.022	RCT
Stress	Scherer, 2016c	Stress	-0.195	0.260	0.068	-0.705	0.315	-0.749	0.454	RCT
Stress	Kamali, 2018	Stress	-0.029	0.233	0.054	-0.486	0.428	-0.126	0.900	RCT
Stress			-0.772	0.276	0.076	-1.313	-0.231	-2.796	0.005	
Overall			-0.571	0.071	0.005	-0.711	-0.432	-8.029	0.000	

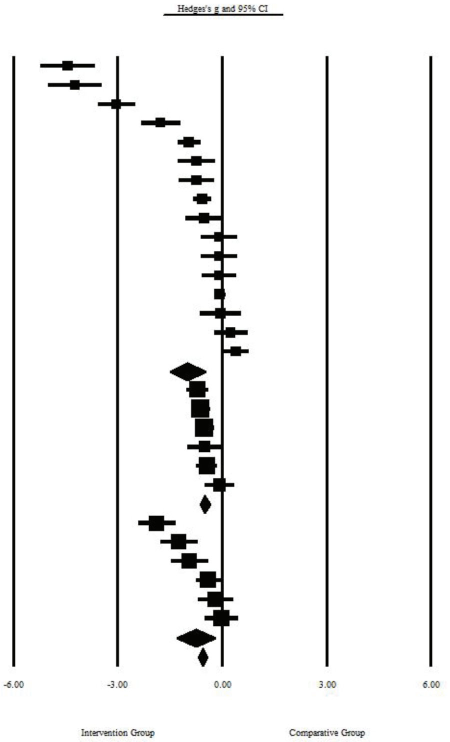

Supplementary Figure 2. Effects of nonpharmacological interventions in randomized controlled trials (RCTs) on women with high-risk pregnancies.
