# Supplementary material for: Effects of nonpharmacological interventions on the psychological health of high-risk pregnant women: a systematic review and meta-analysis
Source: Korean J Women Health Nurs. 2021 Sep 30;27(3):180–95. doi: 10.4069/kjwhn.2021.09.17 (PMC9328588; doi:10.4069/kjwhn.2021.09.17)
Supplement: Supplementary Figure 3. — Effects of nonpharmacological interventions in non-randomized controlled trials (non-RCT) on women with high-risk pregnancies. [file kjwhn-2021-09-17-suppl6.pdf]

Group by Outcome	Study name	Outcome	Hedges's g	Standard error	Variance	Lower limit	Upper limit	Z-Value	p-Value	
Anxiety	Cai, 2020b	Anxiety	-2.561	0.213	0.046	-2.980	-2.143	-11.997	0.000	Non-RCT
Anxiety	Cai, 2020a	Anxiety	-2.381	0.208	0.043	-2.788	-1.974	-11.468	0.000	Non-RCT
Anxiety	Chang S-B, 2009	Anxiety	-1.473	0.330	0.109	-2.121	-0.826	-4.462	0.000	Non-RCT
Anxiety	Yu W-J, 2010a	Anxiety	-1.214	0.278	0.077	-1.759	-0.670	-4.370	0.000	Non-RCT
Anxiety	Asghari, 2016a	Anxiety	-1.165	0.274	0.075	-1.702	-0.628	-4.253	0.000	Non-RCT
Anxiety	Choi MS, 2010b	Anxiety	-0.985	0.282	0.080	-1.538	-0.431	-3.488	0.000	Non-RCT
Anxiety	Choi MS, 2010a	Anxiety	-0.971	0.282	0.079	-1.523	-0.418	-3.443	0.001	Non-RCT
Anxiety	Kim Y-S, 2019a	Anxiety	-0.574	0.302	0.091	-1.167	0.019	-1.898	0.058	Non-RCT
Anxiety	Baek ES, 2013a	Anxiety	-0.547	0.328	0.108	-1.190	0.096	-1.668	0.095	Non-RCT
Anxiety	Kim HJ, 2020	Anxiety	0.542	0.262	0.069	0.029	1.055	2.069	0.039	Non-RCT
Anxiety			-1.142	0.318	0.101	-1.765	-0.519	-3.591	0.000	
Depression	Asghari, 2016b	Depression	-0.828	0.264	0.070	-1.345	-0.311	-3.141	0.002	Non-RCT
Depression	Baek ES, 2013b	Depression	-0.518	0.327	0.107	-1.159	0.124	-1.581	0.114	Non-RCT
Depression	Kim Y-S, 2019b	Depression	-0.368	0.299	0.089	-0.954	0.217	-1.233	0.218	Non-RCT
Depression	Ko JM, 2014	Depression	-0.064	0.240	0.058	-0.534	0.406	-0.265	0.791	Non-RCT
Depression	Dalfra, 2009a	Depression	0.135	0.141	0.020	-0.141	0.412	0.959	0.338	Non-RCT
Depression			-0.287	0.193	0.037	-0.664	0.091	-1.489	0.136	
Stress	Yu W-J, 2010b	Stress	-1.447	0.287	0.082	-2.009	-0.884	-5.040	0.000	Non-RCT
Stress	Choi MS, 2010d	Stress	-0.912	0.280	0.078	-1.461	-0.363	-3.256	0.001	Non-RCT
Stress	Choi MS, 2010c	Stress	-0.781	0.276	0.076	-1.323	-0.240	-2.827	0.005	Non-RCT
Stress	Park H-J, 2017	Stress	-0.708	0.341	0.116	-1.377	-0.040	-2.076	0.038	Non-RCT
Stress	Oh J, 2007	Stress	-0.400	0.246	0.060	-0.881	0.082	-1.627	0.104	Non-RCT
Stress	Dalfra, 2009c	Stress	0.244	0.142	0.020	-0.034	0.521	1.720	0.085	Non-RCT
Stress	Dalfra, 2009b	Stress	0.305	0.142	0.020	0.027	0.583	2.151	0.032	Non-RCT
Stress			-0.493	0.252	0.064	-0.988	0.002	-1.953	0.051	
Overall			-0.509	0.138	0.019	-0.780	-0.239	-3.692	0.000	

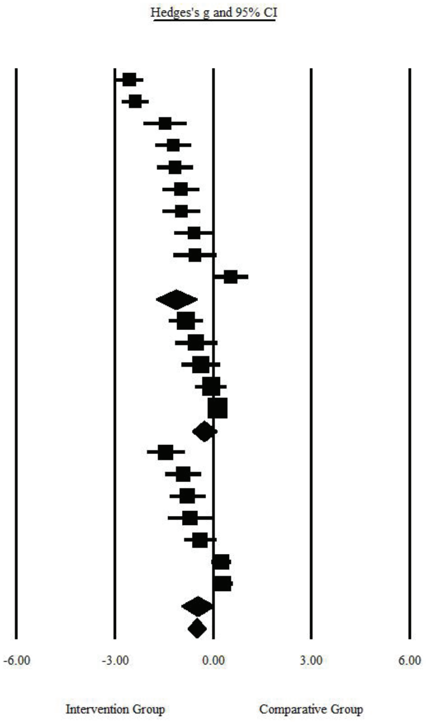

Supplementary Figure 3. Effects of nonpharmacological interventions in non-randomized controlled trials (non-RCT) on women with high-risk pregnancies.

https://doi.org/10.4069/kjwhn.2021.09.17
